# Success of Senegal's first nationwide distribution of long-lasting insecticide-treated nets to children under five - contribution toward universal coverage

**DOI:** 10.1186/1475-2875-10-86

**Published:** 2011-04-13

**Authors:** Julie I Thwing, Robert T Perry, David A Townes, Mame Birame Diouf, Salif Ndiaye, Moussa Thior

**Affiliations:** 1 Malaria Branch, Center for Global Health, Centers for Disease Control and Prevention, Atlanta, USA; 2 National Malaria Control Programme, Dakar, Senegal; 3 Centre de Recherche pour le Développement Humain, Dakar, Senegal

## Abstract

**Background:**

In 2009, the first national long-lasting insecticide-treated net (LLIN) distribution campaign in Senegal resulted in the distribution of 2.2 million LLINs in two phases to children aged 6-59 months. Door-to-door teams visited all households to administer vitamin A and mebendazole, and to give a coupon to redeem later for an LLIN.

**Methods:**

A nationwide community-based two-stage cluster survey was conducted, with clusters selected within regions by probability proportional to size sampling, followed by GPS-assisted mapping, simple random selection of households in each cluster, and administration of a questionnaire using personal digital assistants (PDAs). The questionnaire followed the Malaria Indicator Survey format, with rosters of household members and bed nets, and questions on campaign participation.

**Results:**

There were 3,280 households in 112 clusters representing 33,993 people. Most (92.1%) guardians of eligible children had heard about the campaign, the primary sources being health workers (33.7%), neighbours (26.2%), and radio (22.0%). Of eligible children, 82.4% received mebendazole, 83.8% received vitamin A, and 75.4% received LLINs. Almost all (91.4%) LLINs received during the campaign remained in the household; of those not remaining, 74.4% had been given away and none were reported sold. At least one insecticide-treated net (ITN) was present in 82.3% of all households, 89.2% of households with a child < 5 years and 57.5% of households without a child < 5 years. Just over half (52.4%) of ITNs had been received during the campaign. Considering possible indicators of universal coverage, 39.8% of households owned at least one ITN per two people, 21.6% owned at least one ITN per sleeping space and 34.7% of the general population slept under an ITN the night before the survey. In addition, 45.6% of children < 5 years, and 49.2% of pregnant women had slept under an ITN.

**Conclusions:**

The nationwide integrated LLIN distribution campaign allowed household ITN ownership of one or more ITNs to surpass the RBM target of 80% set for 2010, though additional distribution strategies are needed to reach populations missed by the targeted campaign and to reach the universal coverage targets of one ITN per sleeping space and 80% of the population using an ITN.

## Background

Malaria, a blood parasite transmitted by anopheline mosquitoes, remains a major contributor to morbidity and mortality in the developing world, with an estimated 250 million cases and 880,000 deaths worldwide each year, the majority in children < 5 years old in sub-Saharan Africa [1]. Use of insecticide-treated nets (ITNs) has been shown to reduce clinical episodes by approximately 50% and reduces all-cause mortality by 17% [2]. Where community-level ITN coverage is greater than about 60%, a community effect is seen in which non-users receive similar protection to ITN users [3]..

Increasingly malaria-endemic countries in sub-Saharan Africa are conducting large-scale distributions of ITNs for malaria prevention, many through integrated campaigns targeted to children < 5 years, distributing long-lasting insecticide treated nets (LLINs) along with other child health interventions, including vaccinations (measles and/or polio), de-worming (mebendazole) and vitamin A. Many of these countries are now reporting household ownership greater than 50%, decreases in parasite prevalence in children < 5 years, and corresponding decreases in all-cause child mortality thought to be due in large part to the decreasing burden of malaria [1].

In Senegal, a West African country with an estimated 2009 population of 12.2 million, including 2.3 million children < 5 years [4,5], the National Malaria Control Programme (NMCP) objective is to achieve 80% use of ITNs in the general population, with targets focused on children < 5 years and pregnant women [6]. Strategies to increase ITN ownership have included commercial sales, targeted and untargeted subsidized sales, and distribution of LLINs to vulnerable groups.

Large-scale free distribution of LLINs began in 2008, with a sub-national distribution of 678,556 LLINs to children 6-59 months of age during a round of the semi-annual national vitamin A supplementation and deworming campaign, and another of 466,897 LLINs to children < 5 years in areas served by the World Bank-supported Nutrition Enhancement Programme [7,8]. Household ownership of at least one ITN rose from 36.3% in late 2006 to 60.4% in late 2008, and utilization of an ITN the night before the interview rose from 16.4% to 29.2% for children under five, from 17.2% to 28.5% of pregnant women and from 11.9% to 23.0% of the general population [9,10].

With support from its partners, the Senegal Ministry of Health and Prevention conducted its first nationwide LLIN distribution campaign in 2009, distributing approximately 2.2 million LLINs in two phases (Table 1). The goal was to distribute vitamin A, mebendazole and LLINs to at least 85% of children in targeted age groups. Both phases used a door-to-door strategy to deliver a voucher for an LLIN to be redeemed later at a distribution point, together with mebendazole and vitamin A during the first phase. The Senegal campaign also included a number of communications strategies to advertise the campaign and communicate the importance of using ITNs.

**Table 1:** Details of integrated campaign phases

	Phase 1	Phase 2
Dates	June 22-30, 2009	October 9-15, 2009
Area covered	Nationwide, excluding 6 health districts in Dakar*	6 health districts in Dakar
Interventions	LLINs and vitamin A for children 6-59 months, mebendazole for children 11-59 months	LLINs for children 6-59 months
Number of LLINs	1,840,000	340,000

* Six health districts in Dakar were excluded from this phase because of delays in arrival of enough LLINs for the entire country.

While the integration with the vitamin A and mebendazole campaign provided a ready platform for scaling up the distribution of LLINs to children < 5 years, the overall objective in Senegal is to attain universal coverage of ITNs (access to and use of ITNs among the entire population in all age groups). The contribution of campaigns targeting children < 5 years towards reaching universal coverage has not been explored. Though in most sub-Saharan African countries children < 5 years represent only one-fifth of the population or less, the proportion of households with children < 5 years is higher, varying from 52 to 79% in previous studies [11,12].

Though the objective for an increasing number of countries is universal coverage, the indicators to measure progress towards this objective are still under development. Several indicators have been proposed: the proportion of the general population reported to have slept under an ITN the night before the survey visit, the proportion of all household ITNs that were used the night before the survey visit, the overall ratio of population to the number of ITNs, the proportion of houses with at least one net per every two people, and the proportion of houses with all sleeping spaces covered, among others [13]. Most evaluations of campaigns targeting children < 5 years have focused on coverage within that group; to date evaluations in only two countries have reported the proportion of the general population using an ITN after campaigns targeting children [14]. This community-based cross-sectional household survey was conducted after the 2009 national distribution in Senegal in order to evaluate the progress toward universal coverage by targeted campaigns, to define the remaining ITN gaps, and to determine the proportion of eligible children who received the campaign interventions.

## Methods

### Sampling

Using probability proportional to size sampling, 112 clusters (enumeration areas) were selected, eight from each of the 14 regions of Senegal. Two alternate clusters were selected from each region in case a cluster was unavailable (e.g, inaccessible or politically unstable). All households present in a cluster were mapped using GPS-equipped PDAs and a simple random sample of 30 households per cluster was selected as previously described [15], for a projected total of 3,360 households. Up to three visits were made if no one was at home but no replacements were made for absences or refusals. The survey was powered to detect a difference of 15% between regions in the use of ITNs by children < 5 years, based on an expected frequency of ITN use in children < 5 years of 42% (MIS 2008/9), a design effect of 2, an estimated mean of 1.42 children per household, and a 95% participation rate.

### Timing

The survey was conducted from December 11, 2009 to January 4, 2010, three months after the campaign in Dakar and six months after the campaign in the rest of the country. The rainy season in Senegal varies by latitude but generally falls between July and October, with peak malaria transmission during October and November.

### Questionnaire

The questionnaire included a demographic section with questions on the number of household members and sleeping spaces, and a socio-economic section with questions used to construct wealth quintiles. A net roster listed all nets and their characteristics, and a household roster listed all members and visitors with information about bed net use. The primary guardian of each child eligible for the campaign was interviewed about campaign communications and receipt of campaign interventions, though no data were collected on receipt of vitamin A and mebendazole among children in Dakar. Additional modules asked about old nets not currently in use and about household visits to deliver behaviour change communication messages.

### Definitions

A household was defined as all individuals who eat out of one pot, including guests. An LLIN was defined as a bed net recommended as an LLIN by WHOPES [16], and an ITN was defined as an LLIN, a bed net in use for less than one year that was treated with an insecticide during manufacture but was not long-lasting, or a net treated with insecticide in the past 12 months. A net was defined as hanging if it was reported as having been suspended the previous night, whether or not it was hanging at the time of the interview, as people often take their nets down during the day. A person was defined as having slept under an ITN if they were reported to have slept the previous night under a net defined as an ITN.

### Data management and analysis

Questionnaires were programmed using Visual CE (Visual CE 11.0; Syware Inc., Cambridge, MA, USA) on personal digital assistants (PDAs) and interviews were conducted using these PDAs. At the end of the survey, data were imported to and analysed with SAS 9.2 (SAS Institute, Cary, NC, USA). The SURVEYFREQ procedure was used for analysis of categorical variables and the SURVEYMEANS procedure for analysis of continuous variables, weighted by household, and accounted for clustering by enumeration area. Pre-campaign ITN ownership was determined by subtracting LLINs received from the 2009 campaign from the total number of ITNs in the household. Due to the structure of the questionnaire administered on PDA, children were not linked to specific guardians in the household roster, and if guardians reported no campaign participation, they were not asked about campaign participation by their children. Children listed on the household roster and eligible to participate in the campaign but for whom no data on campaign participation existed, and who lived in households in which one or more guardians reported no campaign participation, were recorded as having not received any campaign interventions. After this assignment was made, there were 396 children eligible for the campaign for whom data on campaign participation were missing.

To determine wealth quintiles, a principal components analysis of 30 elements was performed, including ownership of durable assets (e.g. mobile phone, fixed telephone, radio, stove, refrigerator, bicycle, motorcycle, motor vehicle, horse cart), livestock, housing materials (e.g. roof, wall, floor, window screens), number of rooms and sleeping spaces, and type of access to water, fuel, electricity, and sanitation [17]. The first principal component was used to rank the households.

### Ethical approval

The protocol was reviewed and approved by both the National Ethics Committee for Health Research at the Ministry of Health and Prevention (Comité National d'Ethique pour la Recherche en Santé) and the CDC Human Subjects Committee.

## Results

### Demographics

**Figure 1:**
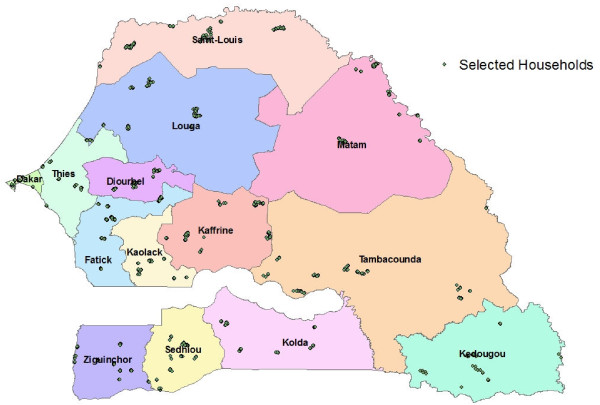
**Clusters selected for the survey**.

In all, 3,315 households in 112 clusters were visited for the survey (Figure 1), with 3280 (98.9%) agreeing to participate. These households included 33,993 individuals, 12,755 nets, 4,049 guardians, and 6,419 children eligible for the campaign. Of all households, 45.9% were urban and 54.1% were rural, and 78.4% of households had at least one child < 5 years. Males represented 47.3% and females represented 52.7% of individuals. There was a median of 7.9 (mean 9.8) regular members per household, and a median of 4.3 (mean 5.7) sleeping spaces.

### Campaign logistics

Of guardians of children aged 6-59 months during the campaign, 92.1% (95% CI 89.8-94.3) had heard about the campaign, 33.7% from a community health agent, 26.2% from a neighbour, and 22.0% from the radio (respondents could select more than one answer). While the majority of guardians reported that mobile teams distributed vitamin A, mebendazole, and a voucher at home, substantial minorities of guardians reported going to the distribution points for all campaign interventions, receiving LLINs directly at home along with the other campaign interventions, and neither receiving a team visit nor going to a distribution point (Table 2). Guardians in urban areas were more likely than guardians in rural areas to report neither receiving a visit nor going to a distribution point, while guardians in rural areas tended to be more likely to go directly to a distribution point. Of guardians who received vouchers and went to distribution points, 87.7% (95% CI 83.3% - 92.1%) reported it was in their village or neighbourhood, 96.4% (95% CI 94.8-97.9) went on foot, and 88.8% (95% CI 85.4 - 92.2) took less than 30 minutes to travel from their homes to the distribution point.

**Table 2:** Campaign distribution methods employed: proportions of guardians of eligible children reporting the distribution method that reached them and those not reached

	Urban % (95% CI)	Rural % (95% CI)	Total % (95% CI)
Received home visit for interventions, including voucher for LLIN	58.7% (48.1-69.4)	58.6% (48.9-68.2)	58.6% (51.2-66.0)
Received all interventions, including LLIN, at home visit	3.7% (1.6-5.8)	6.0% (3.5-8.6)	5.2% (3.4-7.0)
Required to go to distribution post to receive all interventions	17.5% (11.6-23.5)	27.6% (20.1-35.1)	24.2% (18.8-29.6)
No home visit and did not go to distribution post	19.0% (11.0-27.1)	7.5% (5.4-9.6)	11.4% (7.8-15.0)

### Vitamin A and Mebendazole

Of all children living outside Dakar, aged 6-59 months for vitamin A and 12-59 months for mebendazole, 83.8% (95% CI 80.4-87.2) received vitamin A and 82.4% (95% CI 78.9-85.9) received mebendazole. Of children who did not receive these interventions, almost three-fifths were in households in which a guardian reported neither a campaign home visit nor going to the distribution post, and almost one-fifth were absent on the day of the campaign (Table 3).

**Table 3:** Among children who did not receive campaign interventions, reasons why not

	Vitamin A % (95% CI)	Mebendazole % (95% CI)	LLIN % (95% CI)
Child was absent	18.5% (12.8-24.1)	18.0% (12.6-23.5)	11.2% (7.1-15.3)
Parent/guardian refused	1.1% (0.1-2.2)	0.9% (0.0-1.9)	0.1% (0.0-0.3)
Campaign distribution team ran out	7.4% (1.9-12.8)	7.3% (2.0-12.6)	10.1% (6.4-13.9)
Lost/destroyed coupon	---	---	1.1% (0.0-2.6)
Coupon not exchanged for LLIN	---	---	3.9% (1.8-5.9)
No home visit/did not go to distribution post	59.1% (51.0-67.1)	57.4% (49.4-65.5)	55.2% (46.9-63.5)
Don't know/other	14.0% (8.7-19.2)	16.0% (10.6-21.3)	16.6% (12.1-21.1)

### Vouchers and LLINs

The great majority of vouchers received were exchanged for an LLIN (97.9%, 95% CI 97.0-98.8); 47.5% of vouchers not exchanged were due to shortage of LLINs at the distribution point. Including all methods of LLIN distribution, 75.4% (95% CI 70.3-80.4) of children 6-59 months at the time of the campaign received an LLIN from the campaign. There was no disparity by urban/rural zone or by wealth quintile, though there was some regional variation (55.3-89.1%). Children under six months of age at the time of the campaign were not eligible to receive an LLIN; at the time of the survey in regions other than Dakar these children were 6 to 12 months old. However, children < 12 months were no less likely to sleep under an ITN than children 12-59 months (46.2%, 95% CI 39.0-53.3 vs. 45.4%, 95% CI 38.9-52.0). Of nets received during the campaign, 91.4% (95% CI 89.5-93.3) were reported to have remained in the household. Of those not remaining in the household, 74.4% (95% CI 67.4-81.5) were reported given to a friend or neighbour; none were reported sold.

### Household ITN ownership after the campaign

After the campaign, 82.3% of all households owned 1 or more ITNs, 89.2% of households with one or more children < 5 years and 57.5% of households without a child < 5 years. Of all households, 56.3% had one or more ITNs prior to the campaign and 26.3% received their first ITN during the campaign (Table 4). A greater proportion of rural households owned one or more ITNs both before and after the campaign than urban households. In rural households with one or more children < 5 years, 94.6% owned at least one ITN. When regions that received in 2008 a quantity of LLINs equal to the estimated number of children <5 years were compared to regions that did not receive any free LLINs in 2008, the significant difference in the proportion of households owning at least one ITN before the 2009 campaign (65.6%, 95% CI 61.0-70.2 vs. 49.2%, 95% CI 41.5-56.9) disappeared post-campaign (89.7%, 95% CI 86.1-93.2 vs. 85.6%, 95% CI 82.2-89.0). While the campaign almost doubled household ownership of at least one ITN in the capital city of Dakar (32.3% to 64.0%), ITN ownership in Dakar remained lower than the remainder of the country.

**Table 4:** Household ITN ownership, before and after the 2009 integrated campaign

	Urban % (95% CI)	Rural % (95% CI)	Total % (95% CI)
HH owning ≥ 1 ITN prior to the campaign	45.6% (35.6-55.7)	64.3% (60.1-68.5)	55.8% (49.4-62.2)
HH receiving ≥ 1 ITN during the campaign	49.6% (43.2-56.0)	72.2% (68.2-76.1)	61.8% (56.6-67.1)
HH receiving their first ITN during the campaign	26.6% (21.1-32.1)	26.8% (23.6-30.1)	26.7% (23.7-29.8)
HH owning ≥ 1 ITN, post-campaign	72.3% (64.4-80.2)	90.8% (88.6-93.1)	82.3% (77.2-87.5)
HH owning ≥ 1 ITN, among HH with ≥ 1 child < 5 years, post-campaign	81.4% (76.2-86.7)	94.6% (92.7-96.5)	89.2% (85.9-92.5)
HH owning ≥ 1 ITN, among HH without a child < 5 years, post-campaign	50.3% (38.6-62.0)	69.8% (62.3-77.2)	57.5% (48.0-67.1)

HH: Household

### Characteristics of nets present

After the campaign, 79.9% (95% CI 76.7-83.2) of all nets in households were LLINs and 87.5% (95% CI 85.2-89.8) were ITNs. These characteristics did not vary by urban/rural zone or by wealth quintile. Almost three quarters of ITNs, 73.6% (95% CI 70.3-76.9), were received free of charge, 17.4% (95% CI 14.6-20.2) were subsidized, and only 4.4% (95% CI 3.5-5.4) were purchased at full price. The distribution by price paid did not vary by urban/rural zone or by wealth quintile. Most nets had been recently acquired; 75.7% of all nets and 81.9% of ITNs had been acquired in the last 18 months. Just over half (52.4%) of all ITNs were acquired from the campaign. Of those not acquired from the campaign, 40.3% came from health facilities, 31.6% were purchased from merchants, and 8.2% were gifts from family and friends. Two thirds (66.6%, 95% CI 59.9-72.1) of ITNs present in the household were in use the night before the survey with 51.8% (95% CI 48.8-54.8) of hanging ITNs having been received from the campaign. Of those who slept under an ITN, 57.0% (95% CI 54.0-60.0) of the general population, 77.5% (95% CI 74.4-80.6) of children < 5 years and 60.5% (95% CI 53.2-67.9) of pregnant women slept under an LLIN received during the campaign.

### Progress to universal coverage

After the campaign, the average household had 0.31 ITNs per member and 0.55 ITNs per sleeping space. Of all households, 39.8% (95% CI 36.4-43.2) possessed at least one ITN for every two household members, and 21.6% (95% CI 18.0-25.2) had at least one ITN per sleeping space. In regions that received in 2008 a quantity of LLINs equal to the estimated number of children <5 years, the proportion of households owning at least one ITN per two members increased from 11.3% (95% CI 8.7-13.9) before the 2009 campaign to 37.9% (95% CI 34.0-41.8) after the campaign. In regions that did not receive any free LLINs in 2008, the proportion of households owning at least one ITN per two members increased from 6.4% (95% CI 3.3-9.4) before the 2009 campaign to 33.5% (95% CI 29.5-37.5) after the campaign. Overall, 34.7% (95% CI 29.3-40.1) of the general population, 45.6% (95% CI 39.1-52.1) of children < 5, and 49.2% (95% CI 42.5-56.0) of pregnant women slept under an ITN the night before the survey.

### Equity analysis

Households in the lowest socioeconomic quintile had more sleeping spaces (mean 7.2, 95% CI 6.4-8.1) and more members (mean 11.7, 95% CI 10.5-12.9) than households in the highest socioeconomic quintile (mean 5.2 sleeping spaces, 95% CI 4.8-5.6; mean 8.5 members, 95% CI 7.8-9.3). Prior to the campaign, households in the lowest quintile were more likely to own one or more ITNs than households in the highest quintile, and a greater proportion of households in the lower quintiles received LLINs during the campaign, resulting in higher post-campaign ownership of one or more ITNs in the lowest quintile than the highest (Table 5). However, when analysed based on the adequacy of intra-household access, defined as at least one ITN per two members, this relationship disappears. Indeed, while the difference misses statistical significance, the highest quintile trends toward having a greater proportion that meets the goal of one ITN per two people than the lowest quintile, despite having a lower proportion that own one or more ITNs.

**Table 5:** Equity analysis of pre and post campaign ITN ownership, campaign LLIN distribution, and progress toward universal coverage

Quintile	1 (lowest) % (95% CI)	2 % (95% CI)	3 % (95% CI)	4 % (95% CI)	5 (highest) % (95% CI)
Pre-campaign HH ITN ownership	67.5% (60.6-74.5)	68.1% (62.2-74.0)	61.3% (55.3-67.3)	60.4% (52.7-68.0)	43.7% (33.9-53.5)
Eligible HH received LLIN during campaign	84.0% (78.3-89.7)	86.0% (81.1-90.9)	80.4% (74.8-86.0)	79.9% (73.1-86.6)	64.4% (56.4-72.4)
Post-campaign HH ITN ownership	91.5% (88.2-94.9)	92.8% (90.5-95.1)	88.0% (84.4-91.6)	81.1% (72.9-89.4)	73.5% (65.5-81.6)
HH with ≥ 1 ITN per two members	32.0% (27.1-36.8)	34.1% (28.7-39.5)	40.4% (35.2-45.5)	43.8% (37.6-50.0)	42.5% (36.1-49.0)

Of those households lacking nets of any kind, 37.1% overall reported that they had no means to acquire a net. In the lowest quintile, 83.2% (95% CI 70.3-96.1) reported that they had no means to purchase a net, while in the highest quintile, 35.2% (95% CI 23.9-46.6) said they use something else, and 19.7% (95% CI 9.1-30.4) said it was not necessary.

## Discussion

Senegal's first nationwide free distribution of LLINs coupled with mebendazole and vitamin A reached 75% of children < 5 with an LLIN, and over 80% of children < 5 with vitamin A and mebendazole. Though falling slightly short of its objective of reaching 85% of targeted children, the campaign increased overall household ownership of at least one ITN to over 80%. This campaign was different from previous nationwide campaigns reported in the literature [11,12,18-20] in that it was not integrated with a vaccination campaign; however, it appears to have had comparable results in terms of coverage of eligible children. Despite concerns that omitting distribution to children under six months would leave these children unprotected, an apparent redistribution in and among households covered these vulnerable children after the campaign.

The majority of children who did not receive the campaign interventions were those whose guardians reported neither receiving a visit at home nor going to a distribution point, followed by children who were not present at the time of the campaign. While the door-to-door distribution of vouchers for later redemption of an LLIN reached a majority of households, almost one-third of caretakers reported receiving campaign interventions through different strategies, either receiving all interventions simultaneously at the house, or more frequently, being requested to go to a central distribution point for all interventions. Of those that received vouchers door-to-door, almost all went to a distribution point to get an LLIN, and were able to exchange their vouchers. The distribution points appeared to have been well-placed, with most respondents reporting a distribution point in their villages, travelling on foot to arrive in under 30 minutes.

Although there have been anecdotal reports from some countries of campaign LLINs being sold in the market or unused after campaigns, in Senegal the overwhelming majority were reported to have remained in the household and were being used. Of the few that did not remain in homes, most were reported given away to family or friends; not one was reported sold, consistent with what was seen in Niger [18].

While this campaign targeted children 6-59 months of age, Senegal has a policy of universal coverage, or high access to and use of ITNs by all age groups. Of ownership indicators of universal coverage, just under 40% of households have at least one ITN for every two people, and approximately 20% have one ITN per sleeping space. Despite high household ownership of at least one ITN, households still own only half the number of ITNs needed to cover every sleeping space. The results suggest that regions that had not had previous campaigns largely "caught up" with regions that benefitted from previous LLIN distributions in terms of household ownership of at least one ITN and partially caught up in terms of ratio of ITNs to household members or sleeping spaces. While households in rural areas with one or more children < 5 have exceedingly high household ITN ownership, household ITN ownership is lower in urban areas and in households without children < 5. The equity analysis demonstrates the continued importance of providing free LLINs. Though the lowest quintile has a higher proportion of households owning at least one ITN, they have fewer ITNs per household member than the highest quintile. In addition, the highest quintile largely overlaps with the urban population, where families state they rely on screens, fans, mosquito coils, and other non-ITN interventions, while the rural poor rely principally on nets. A review by Kilian of published and gray literature evaluating LLIN distribution strategies found that high household ITN ownership results in high equity, with campaigns achieving rapid increases in net coverage and increases in equity of net ownership [13].

Utilization rates for ITNs were relatively low given the high level of ownership in comparison with other reports [11,12,14,18-21]. While the proportion of houses with at least one ITN is high, given the large median household size, only a minority of households have enough ITNs to allow every member to sleep under one. Also, both this survey and the previous Senegal Malaria Indicator Survey were conducted at the beginning of the dry season, when usage has been noted to be lower [22]. In Niger, ITN usage during the dry season immediately after the campaign was found to be three-fold lower than during the following rainy season, and in Togo, dry season usage was also significantly lower than rainy season, though not as strikingly [11,18].

This study had a number of limitations. As noted above, the reported rates of utilization may be low because the survey was conducted during the dry season. While not capturing optimal usage, this timing still allows for an assessment of effectiveness of the campaign in reaching its distribution targets, progress toward goals in ITN ownership, and equity of ITN ownership. As with most other surveys examining ITN use, there was no verification as to whether the person in question actually slept under the ITN the night before the survey. Though in a few cases, the existence of the net in question was not verified, people may be more likely to under-report nets in hope of being given another, underestimating ownership. While the vast majority of vouchers were reported exchanged for an LLIN, and most LLINs were reported to remain in the homes of the recipients, this was self-reported, and may have been prone to recall or reporting bias. Data on campaign participation was missing for 6.2% of all children, however the proportion of these children who slept under nets acquired from the campaign was similar to children for whom data was available, thus it is unlikely that the missing data biased the results.

Senegal is now embarking on a nationwide rolling campaign targeting universal coverage, distributing one LLIN per sleeping space. The results of this campaign evaluation have been fundamental in the planning of the universal coverage campaign. At the same time, the NMCP is strengthening behaviour change communication programs to ensure people use the nets they have available. These efforts hopefully will close the remaining gaps in ITN possession and use in Senegal and enable the country to achieve truly universal coverage.

## Conclusions

Senegal's first nationwide LLIN distribution placed it on the small list of African countries that have succeeded in reaching the RBM target of 80% of households owning at least one ITN. However, a strategy targeting children < 5 years has not resulted in similar proportions of households achieving universal coverage targets, even in regions having such campaigns two years in a row. The number of nets per household did not cover all sleeping spaces in the majority of households, and in urban areas, where fewer households have children < 5 years, a lower proportion of households own an ITN. Additional strategies, such as the ongoing campaign to distribute LLINs based on the number of sleeping spaces, will be necessary to reach households not yet reached by campaigns targeting children < 5 years. As the scale-up of LLINs continues, the proposed indicators for universal coverage will need to be refined and ultimately compared to measures of disease impact to ensure that they can guide program managers and the international community in the correct choice of strategies.

## Competing interests

The authors declare that they have no competing interests.

## Authors' contributions

JT wrote the protocol, programmed the PDAs, trained and supervised the enumerators, analysed the data, and wrote the manuscript. RP participated in implementation of the campaign, wrote the protocol, oversaw logistics, and assisted with data cleaning, analysis, and manuscript writing. DT assisted with PDA programming, training and supervision, and manuscript writing. MBD was involved with implementation of the campaign, writing the protocol, and the manuscript. SN assisted with development of the protocol, and was primarily responsible for training and supervision of enumerators, as well as all field logistics. MT oversaw campaign implementation and involved with protocol development and manuscript writing. All read and approved the final manuscript.
